# The Correlation Between Delayed Surgical Therapy After the Diagnosis of Pilonidal Sinus Disease and Relapse, Labor Loss, and Costs

**DOI:** 10.7759/cureus.6255

**Published:** 2019-11-28

**Authors:** Turgay Karatas, Murat Kanlioz

**Affiliations:** 1 Anatomy, Inönü University, Malatya, TUR; 2 General Surgery, Beylikdüzü Kolan Hospital, Istanbul, TUR

**Keywords:** pilonidal sinus disease, relapse, symptom duration, length of hospital stay, cost

## Abstract

Introduction

There has been no detailed study of the relationship between recurrence and symptom duration in pilonidal sinus disease. The aim of this study was to explore the correlation between delayed surgical therapy after symptoms appear in sacrococcygeal pilonidal sinus disease (SPSD) and relapse, labor loss and costs.

Methods

Patients diagnosed with SPSD were split into two groups according to symptom duration before surgery: 30 days or less (G1) and more than 30 days (G2). Patients included those who underwent Limberg flap reconstruction (LFR) for primary SPSD. The length of hospitalization during surgery, postoperative follow-up period, and, if any, relapse periods were obtained from patients' files. Of the patients with incomplete information, only those whose information was confirmed by phone were also covered by the study.

Results

G1 included 153 patients, including 37 (24.18%) females and 116 (75.82%) males. The median age in G1 was 22 years and the mean age was 23.08±8.72 years. G2 included 214 patients, including 51 (23.83%) females and 163 (76.17%) males. The median age in G2 was 22 years and the mean age was 22.64±9.06 years. The mean lengths of hospitalization in G1 and G2 were 2.14±0.86 and 2.98±1.04 days, respectively, and the difference between them was significant (p<0.03). The follow-up periods in G1 and G2 were 61.52±29.84 (12-108) and 64.0±31.24 (12-113) months, respectively. Relapse occurred in nine patients (5.8%) in G1 and 19 patients (8.8%) in G2, and the difference between them was significant (p<0.02). The mean relapse period was 3.44±6.01 and 11.23±7.62 months in G1 and G2, respectively, and the difference between them was significant (p<0.04).

Conclusion

Delayed surgery should be avoided to minimize the costs and the probability of relapse in SPSD.

## Introduction

Therapy for pilonidal sinus disease (PSD) has remained controversial since it was first introduced by Hodges in 1880 [1]. Sacrococcygeal pilonidal sinus disease (SPSD) is an infectious disease characterized mostly by sinus orifice(s) in the midline, approximately 5 cm from the anus, seen in the "natal cleft" and sacrococcygeal region [2]. Though the debate on whether it is congenital or acquired has been ongoing for a long time, it is today widely accepted that the disease is acquired (3). The disease occurs at an earlier age in women, mostly between 20 and 25 years of age. The female-to-male ratio ranges from 1:3 to 1:5 [3-4]. It is common knowledge that prolonged sitting, chronic trauma to the sacrococcygeal region, oily skin, hirsutism, and obesity are among the factors increasing the occurrence of SPSD [5-6]. Beyond television, screen dependency has today been further strengthened by the outcomes of the developments in computer technologies, namely, phones, tablets, and computers [7]. In conjunction with the screen dependency, a sedentary lifestyle involving prolonged sitting results in obesity and increased SPSD rates. SPSD can be treated both medically and surgically. However, numerous papers suggest that surgery is the golden standard in its therapy, which is also supported by the results of the therapy [3-4]. In this study, we aimed to identify whether the time elapsed from symptom onset to surgery correlated with the length of hospital stay for surgery and the relapse.

## Materials and methods

The study included patients who underwent Limberg flap reconstruction (LFR) in primary SPSD from 2006 to 2018. Those who were covered by the study included patients who were followed up postoperatively for at least a one-year period. By selecting all patients from the group, including those who underwent LFR, we intended to homogenize the groups and to more effectively evaluate the efficacy of the therapy. The patients who were admitted to our clinic once diagnosed with SPSD and underwent LFR were split into two groups according to the duration of symptoms. Those symptoms were defined as pain, swelling, and discharge in the sacrococcygeal region. Patients included in the first group (G1) had symptom duration of 30 days or less. In the second group (G2), patients had symptom duration of more than 30 days. We examined the patients based on the information available in the patients' files and follow-up forms and recorded the duration of symptoms until surgery, length of hospital stay, whether relapse occurred, and, if it occurred, the time elapsed from surgery to the occurrence of relapse. We contacted patients with incomplete information by phone and confirmed whether relapse occurred and, if it had, how much time elapsed between the surgery and the occurrence of relapse. We then compared the patients in G1 and G2 in terms of relapse rates and the length of hospital stay. The distribution between the two groups was analyzed using the Kolmogorov-Smirnov test. Further, the correlation between the groups was evaluated using the Chi-square test, in which p<0.05 was considered significant. We also used SPSS statistical software (IBM Corp., Armonk, NY) for recording and analyses.

## Results

The median age in G1 was 22 years and the mean age was 23.08±8.72 years, whereas the G2 group included 153 patients, of which 37 (24.18%) were female and 116 (75.82%) were male. The median age in G2 was 22 years and the mean age was 22.64±9.06 years, and the group included 214 patients, of which 51 (23.83%) were female and 163 (76.17%) were male. No significant difference was found between the demographic data of the patients in both groups. In G1 and G2, the patients' mean length of hospital stay was 2.14±0.86 days and 2.98±1.04, respectively, which totaled 2.63±0.96 days (G1 + G2). The difference between G1 and G2 was statistically significant in terms of the mean lengths of hospital stay (p<0.03). The mean follow-up period after surgery (LFR) was 61.52±29.84 months (12-108 months) in G1 and 64.01±31.24 months in G2 (12-113 months). During those follow-up periods, relapse occurred in nine (5.8%) patients in G1, of which two were female (5.4%) and seven were male (6%). In G2, however, relapse occurred in 19 (8.8%) patients, of which five (9.8%) were female and 14 (8.5%) were male. The relapse rates of patients in G1 were higher in males, and there was no statistical significance between them (p=0.07). In contrast, the relapse rates of G2 patients were higher in females, and the difference between them was statistically significant (p<0.05). When we compared G1 and G2, we found a higher rate of relapse in G2 and the difference between them was significant (p<0.02). The mean relapse period was found to be 13.44±6.01 months in G1 and 11.23±7.62 months in G2. In G2, relapse occurred earlier than in G1 and the difference between the two groups was statistically significant (p<0.04). Also, in both groups, about 68% of relapses occurred in the first year. Six (67%) out of nine relapses in G1 and 13 (68%) out of 19 relapses in G2 occurred in the first year (Table 1).

**Table 1:** Length of hospital stay and relapse rates after LFR according to the duration of SPSD symptoms LFR: Limberg flap reconstruction; SPSD: sacrococcygeal pilonidal sinus disease

	G1		G2		G1 (+) G2	
	Female	Male	Female	Male	Female	Male
Number of Patients (n)	37 (24.18%)	116 (76.82%)	51 (23.83%)	163 (76.17%)	88 (23.98%)	279 (76.02%)
Total Number of Patients (n)	153		214		367	
Median Age (year)	22		22		22	
Mean Age (year)	23.08±8.72		22.64±9.06		22.82±8.91	
Mean Length of Hospital Stay (day), *p (statistically significant)	2.14±0.86		2.98±1.04		2.63±0.96	
	*p<0.03					
Mean Follow-up Period (month)	61.52±29.84		64.01±31.24		62.97±30.65	
Min.-Max. Follow-up (month)	12-108		12-113		12-113	
Number (n) and rate (%) of the patients in which relapse occurred *p (statistically significant)	Female 2 (5.4%)	Male 7 (6%)	Female 5 (9.8%)	Male 14 (8.5%)	Female 7 (7.9%)	Male 21 (7.5%)
	p<0.07		*p<0.05		p<0.08	
	Total 9 (5.8%)		Total 19 (8.8%)		Total 28 (7.6%)	
	*p<0.02					
Mean Relapse Period (month) and *p (statistically significant)	13,44±6,10		11.23±7.63		11.94±7.14	
	*p<0.04					
The ratio of relapses in the first year to relapses in the total follow-up period (%)	67		68		68	

## Discussion

In the study groups (G1 + G2), the patients' mean age was 22.82±8.91 years, and the female-to-male ratio was approximately 1:3. Erkent et al. reported a mean age of 28.4 years and a female-to-male ratio of approximately 1:4 [8].

As we take a closer look at the results of LFRs performed in SPSD, the study conducted by Demiryas et al. pointed out that the mean relapse rate of patients treated with LFR for SPSD was 6% at a mean follow-up period of 48.2 ± 21.7 months [9]. Another study performed by Boshnaq et al. indicated that the relapse rates of PSDs treated with LFR were 7.7% at an 18-month follow-up period [10]. Milone et al. reported that the overall relapse rate was 13.8% in their meta-analysis covering a follow-up period of 58-240 months, however, the relapse rates increased over 60% in some of their studies at 240-month follow-up periods [11]. In another study remarkable for focusing on the relapse and follow-up periods, Stauffer et al. noted that relapse in PSD depends on both the surgical procedure and the follow-up period. Even though relapse rates seem to be lower in short-term follow-ups, higher rates are reported in long-term follow-ups [12]. In another study that we consider important since it represents a different point of view on the analysis of relapse, Burnett et al. have revealed that many surgeons have a positive perception of the success of therapies they administered since they do not keep abreast of relapses in those cases [13]. About 68% of the relapses in our study occurred in the first year and the difference between G1 and G2 was not significant (p<0.8). Likewise, Halleran et al. reported that most relapses (80%) in 307 cases included in their study occurred in the first year [14].

Another parameter in our study was to assess the length of hospital stay. On the basis of the available data, we concluded that the length of hospital stay was shorter in G1 patients (2.14±0.86 days) in comparison to those in G2 (2.98±1.04 days), and the difference between the two groups was significant (*p<0.03). This outcome suggests to us that a prolonged duration of symptoms, leading to those symptoms becoming complicated, results in a prolonged duration of therapy and increased costs. We have also found in the literature various results on the duration of hospital stay. Koca et al. noted that the length of postoperative hospital stay was 5.98±2.21 days in patients with complicated pilonidal sinus treated with LFR and V-Y flap [15]. In another study, Sewefy et al. stated that the mean length of hospital stay after SPSD surgery was 4.9±2.4 days [16]. Nonetheless, there are also relatively shorter lengths of hospital stay reported in the literature. Bayhan et al. reported that the length of hospital stay in the group treated with modified LFR for SPSD was 1.25±0.4 days [17]. In their study, Zagory et al. indicated that they performed primary closure after pediatric SPSD resection and that the mean length of hospital stay was 0.67 days [18]. An increased length of hospital stay leads to an increase in both costs and labor loss.

The mean follow-up period of the 367 patients treated with LFR was 62.97±30.65 months, and relapse occurred in 28 (7.6%) patients. The relapse rate in all patients was 7.6%, which was 5.8% in G1 patients and 8.8% in G2 patients (*p<0.02). The difference between G1 and G2 in terms of relapse rates suggests to us that a delay in surgery should be avoided in SPSD. At present, there are no similar studies examining the symptom onset period and the occurrence of relapse in the literature.

## Conclusions

Taking into consideration the fact that one of the most important parameters proving success in SPSD therapy is to consider whether relapse has occurred or not and our suggestion to reduce recurrence, a delay in surgery should be avoided in SPSD. As can be seen from the study results, the sooner the surgery is performed, the lower the length of hospital stay and costs.
